# Early-onset lupus nephritis

**DOI:** 10.1093/ckj/sfae212

**Published:** 2024-07-13

**Authors:** Francesco Peyronel, Giovanni M Rossi, Giulia Palazzini, Ludovica Odone, Carmela Errichiello, Giacomo Emmi, Augusto Vaglio

**Affiliations:** Nephrology and Dialysis Unit, Meyer Children's Hospital IRCCS, Florence, Italy; Department of Experimental and Clinical Medicine, University of Florence, Florence, Italy; Nephrology Unit, Parma University Hospital, Parma, Italy; Department of Medicine and Surgery, University of Parma, Parma, Italy; Laboratorio di Immunopatologia Renale “Luigi Migone”, University of Parma, Parma, Italy; Department of Biomedical Experimental and Clinical Sciences “Mario Serio”, University of Florence, Florence, Italy; Nephrology and Dialysis Unit, Azienda Socio-Sanitaria Territoriale (ASST) Papa Giovanni XXIII, Bergamo, Italy; Nephrology and Dialysis Unit, Meyer Children's Hospital IRCCS, Florence, Italy; Department of Medical, Surgery and Health Sciences, University of Trieste, Italy; Clinical Medicine and Rheumatology Unit, Cattinara University Hospital, Trieste, Italy; Centre for Inflammatory Diseases, Monash University Department of Medicine, Monash Medical Centre, Melbourne, Australia; Nephrology and Dialysis Unit, Meyer Children's Hospital IRCCS, Florence, Italy; Department of Biomedical Experimental and Clinical Sciences “Mario Serio”, University of Florence, Florence, Italy

**Keywords:** interferonopathy, lupus nephritis, monogenic lupus, paediatric lupus

## Abstract

Early-onset systemic lupus erythematous (SLE) is a distinct clinical entity characterized by the onset of disease manifestations during childhood. Despite some similarities to patients who are diagnosed during adulthood, early-onset SLE typically displays a greater disease severity, with aggressive multiorgan involvement, lower responsiveness to classical therapies, and more frequent flares. Lupus nephritis is one of the most severe complications of SLE and represents a major risk factor for long-term morbidity and mortality, especially in children. This review focuses on the clinical and histological aspects of early-onset lupus nephritis, aiming at highlighting relevant differences with adult patients, emphasizing long-term outcomes and discussing the management of long-term complications. We also discuss monogenic lupus, a spectrum of conditions caused by single gene variants affecting the complement cascade, extracellular and intracellular nucleic acid sensing and processing, and occasionally other metabolic pathways. These monogenic forms typically develop early in life and often have clinical manifestations that resemble sporadic SLE, whereas their response to standard treatments is poor.

## INTRODUCTION

Systemic lupus erythematosus (SLE) is a chronic autoimmune disease usually affecting adult individuals. However, ∼15% to 20% of all SLE patients are diagnosed during childhood. This disease subset, commonly referred to as ‘paediatric’, ‘childhood-onset’, or ‘early-onset’ SLE, is often considered as a distinct clinical entity because, although its clinical manifestations and immunological markers are similar to those encountered in adults, its phenotype is often more aggressive, and long-term complications also differ. Lupus nephritis (LN) represents one of the most common and severe complications of SLE, being a major risk factor for long-term morbidity and mortality, particularly in children. This review focuses on the clinical and histological aspects of early-onset LN, and highlights relevant differences from adult forms.

## EPIDEMIOLOGY

In the paediatric population, SLE has an estimated prevalence of 1.9–25.7 per 100 000 children and an incidence of 0.3–0.9 per 100 000 children per year [1, 2]. More than 60% of early-onset SLE cases are diagnosed in children between 10 and 18 years of age, whereas the disease onset is seen before the age of 5 years in only 5% of cases [3–6]. Rare cases of neonatal SLE have also been described, usually in association with an active maternal disease [7]. When SLE onset occurs before the age of 8 years, no significant sex disparity is observed, whereas from puberty onwards, it becomes more common in girls, who have a 5- to 9-fold higher incidence rate than boys [3, 8, 9].

As for adult patients, ethnicity also influences the epidemiology and the clinical course of the disease. Indeed, early-onset SLE is more common and aggressive in African-American, Hispanic, and Asian patients than in White patients [8, 10, 11]. Moreover, a worse prognosis has been demonstrated in patients with a lower socio-economic status, even if limited data are available on epidemiology and outcomes from large populations of low- and middle-income countries [12].

The proportion of patients with early-onset SLE who develop nephritis largely varies across studies and, similar to adult cohorts, it is present in roughly 30%–50% of patients [3, 13–15]. The difference in report rates is an unresolved issue that might be due to a series of reasons, including discrepancies in biopsy policies, cohort size, and racial cohort composition. The time of onset of LN in children, however, is consistent across studies, with most patients developing it within 1–2 years after the diagnosis of SLE [3, 13–15]. Another consistent finding is that of a higher frequency of kidney involvement in non-Caucasian vs Caucasian patients (62% vs 45% in one study) [10].

## CLINICAL FEATURES

From a clinical point of view, the disease is characterized by a greater severity at onset, with aggressive multiorgan involvement, lower responsiveness to classical therapies, and more frequent flares, as compared to adults [16–18].

The disease onset is often insidious, with a high number of patients presenting with fever, weight loss, fatigue, and arthralgia, all of which may persist for weeks to months. Major organ involvement typically develops within the first 2–3 years after the appearance of the first symptoms. Nevertheless, in some patients the disease breaks out with very severe and life-threatening manifestations, such as macrophage activation syndrome, neurological involvement, acute thromboembolic disease, and rapidly progressive glomerulonephritis [16, 17, 19]. As a result, paediatric patients tend to have higher SLE Disease Activity Index (SLEDAI) scores than adults [20].

**Figure 1:**
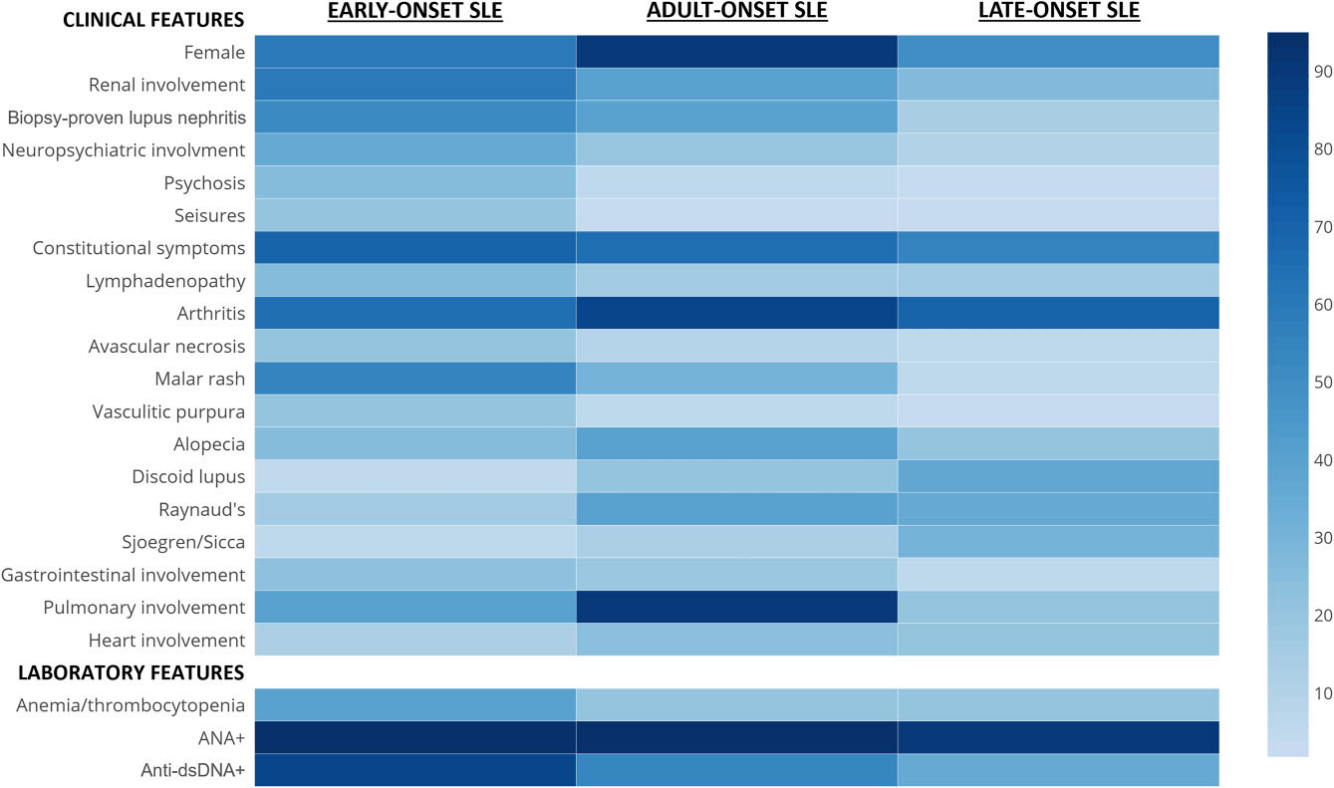
Comparison of clinical features and laboratory findings between early-, adult-, and late-onset SLE. The heatmap highlights the different frequency of individual characteristics and clinical manifestations, as well as of haematological and serological markers, across different age-based subgroups of patients with SLE. The data used to generate this heatmap largely come from references [17, 19, 22–25].

Despite some similarities to patients who are diagnosed during adulthood, some differences in the clinical and serological phenotype of early-onset SLE are evident (Fig. 1). For instance, neurological involvement is more frequent and severe in paediatric patients, particularly in terms of neuropsychiatric manifestations, such as headache, cognitive impairment, mood disturbances, and psychosis [21]. On the other hand, pulmonary involvement, arthritis, Raynaud's phenomenon, photosensitivity, and ‘sicca’ symptoms (i.e. xerophthalmia, and xerostomia) are more common in adult patients [19, 22–25]. Figure 1 also shows the frequency of clinical features in late-onset SLE, i.e. SLE developing in elderly patients. It is interesting to observe that some disease characteristics are similar in children and in elderly patients (e.g. the M:F ratio), while others tend to have frequency and severity that progressively decline with age (e.g. renal and neuropsychiatric involvement). These similarities and differences might be accounted for by several factors, including hormonal status, comorbidities, and the use of drugs.

Similar positivity rates for most circulating autoantibodies are described, particularly when considering anti-nuclear antibodies (ANA). Nevertheless, anti-dsDNA and anti-cardiolipin IgM positivity is more frequent in children, whereas anti-SSA/anti-Ro and anti-SSBB/anti-La are more frequently found positive in adults [24, 26]. Thromboembolic events linked to antiphospholipid antibodies are uncommon in early-onset SLE, and occur more frequently in adults [27].

LN represents one of the most common and severe complications of SLE, being a major risk factor for long-term morbidity and mortality, particularly in children. As for adults, ∼90% of patients with LN have positive anti-dsDNA, while anti-Sm antibodies represent the second most common autoantibody positivity (50%) [3]. In most cases, patients with LN have mild urinary abnormalities (i.e. low-grade proteinuria and/or microscopic haematuria), whereas nephrotic-range proteinuria is found in up to 50% of patients [28, 29]. Acute kidney injury (AKI) due to rapidly progressive glomerulonephritis and/or tubulointerstitial damage is not uncommon at onset. Along the disease course, the estimated prevalence of AKI is 19%–51% [30], with a minority of patients requiring haemodialysis [31]. The development of severe AKI was found to be associated with proliferative lesions at kidney biopsy (i.e. class III or IV, with or without class V) [31].

Among the several classification criteria developed for SLE, the most recent are the ACR/EULAR 2019: these include positive ANA at least once as a required entry criterion, followed by additive weighted criteria grouped in seven clinical (constitutional, haematologic, neuropsychiatric, mucocutaneous, serosal, musculoskeletal, renal) and three immunologic (antiphospholipid antibodies, complement fractions, SLE-specific antibodies) domains, and weighted from 2 to 10. Patients accumulating ≥10 points are classified. In adults, these criteria had a sensitivity of 96% and specificity of 93% [32]. Table 1 shows the performance of these criteria across different studies on childhood-onset SLE. While their sensitivity was usually good, their specificity varied substantially [33–41].

**Table 1:** Performance of the ACR/EULAR 2019 classification criteria in studies on childhood-onset SLE.

					EULAR/ACR–2019	
Author, [reference] year	Country	Mean age at diagnosis (years)	Age range (years)	Number of patients	Sensitivity (%)	Specificity (%)
A.R. Fonseca [33]2019	Brazil	10.6	<18	122	95.1	58.4
M. Ma [34]2020	USA	13.1	<19	156	97.4	98.4
Y. Levinsky [35]2021	Multi-national	13.0	<18	112	96	89
N. Aljaberi [36]2021	USA	15	2–21	112	85	83
R. Abdwani [37]2021	Oman	7.3	<13	133	first visit 81first year 88last visit 89	first visit 92first year 90last visit 90
E.D. Batu [38]2021	Turkey	13.3	0–18	262	91.6	88.5
E.M.D. Smith [39]2021	UK	12.8	<18	482	first visit 94last visit 96	first visit 77last visit 81
A. Ohara [40]2022	Japan	12.8	<16	53	100	84.9
E. Babgi [41]2024	Saudi Arabia	13.9	<14	245	99.2	86.2

## PATHOPHYSIOLOGY AND HISTOPATHOLOGY

**Figure 2:**
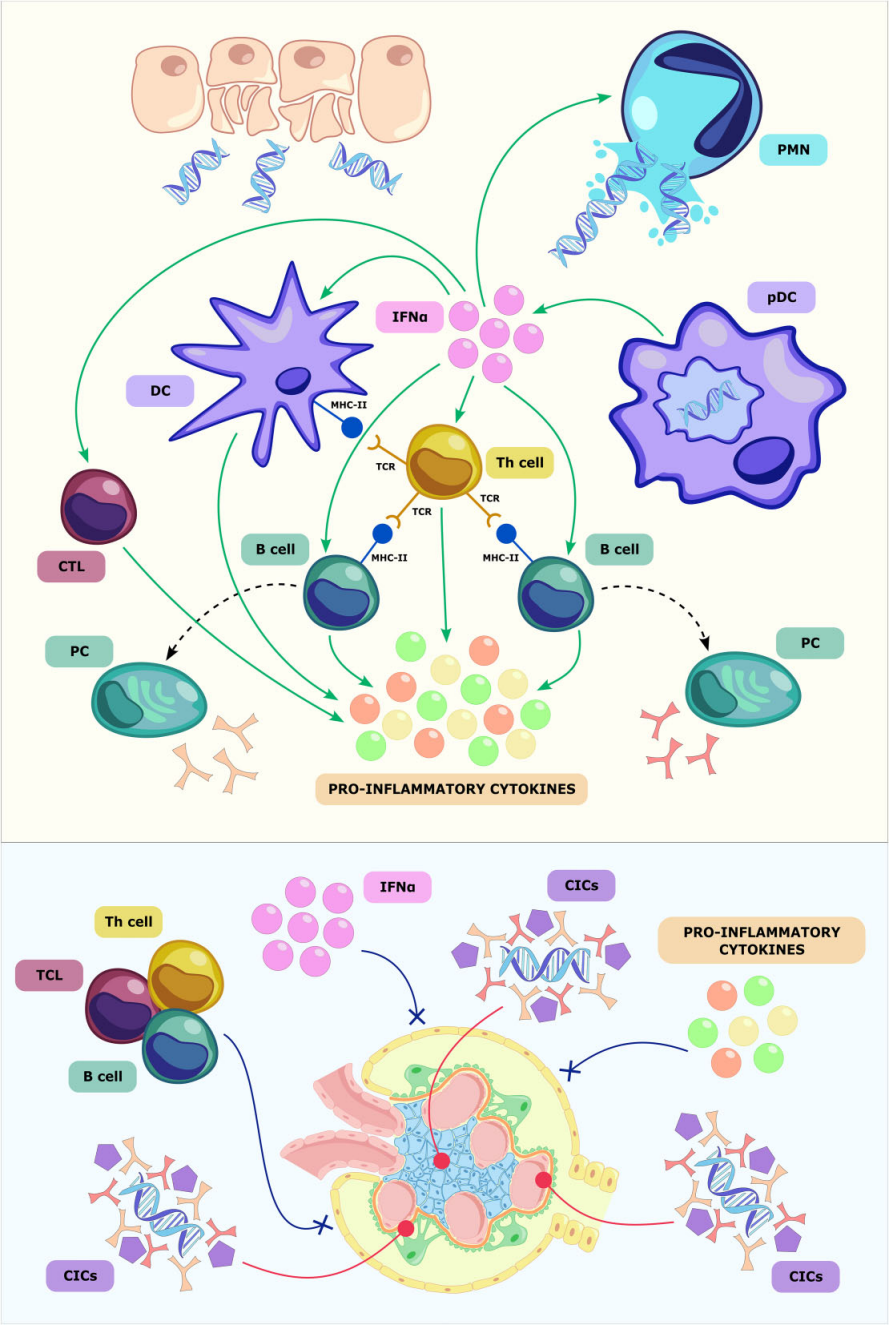
Pathophysiology of LN. Upper panel shows the defects in the clearance of apoptotic debris together with an excessive production of NETs trigger the autoimmune response against nuclear antigens, eventually leading to the production of circulating immune complexes (CICs) composed mainly of autoreactive antibodies capable of binding and activating complement. Autoantigens are captured by plasmacytoid dendritic cells (pDC), which represent the main source of interferon alpha (INFα). IFNα orchestrates the immune response and is responsible for the activation of a broad range of immune cells, including dendritic cells (DC), T helper (Th) cells, cytotoxic T lymphocytes (CTL), and B cells. A variety of cytokines and other stimulating factors induce the maturation of B cells, eventually leading to the generation of autoreactive plasma cells (PC). Lower panel shows that, in LN, glomerular injury is caused by a massive deposition of CICs, usually occurring in the mesangial, subendothelial, and sub-epithelial space (red lines). These induce complement (purple pentagons) activation, which contributes to tissue damage in addition to cytotoxicity deriving from lymphocytes, pro-inflammatory cytokines, and INFα (blue lines), the latter primarily targeting podocytes, endothelial, and parietal epithelial cells. PMN: polymorphonuclear leukocyte; DC: dendritic cell; pDC: plasmacytoid dendritic cell; Th: T helper; CTL: cytotoxic T lymphocyte; PC: plasma cell; IFNα: interferon alpha.

A wide variety of factors play a role in the pathogenesis of SLE and LN. On a background of genetic susceptibility, environmental exposures, and hormonal factors, both an altered clearance of apoptotic cell debris and an impairment in innate and adaptive immunity are crucial. Autoantigens result from the deficient clearance of apoptotic bodies and the prolonged permanence of neutrophil extracellular traps (NETs), eventually triggering an autoimmune response characterized by a crosstalk among different immune cell types, particularly dendritic cells, T cells, and B cells. The activation of B cells and the amplification of the immune response is driven by a broad spectrum of cytokines and stimulating factors, eventually leading to the synthesis of autoreactive antibodies by plasma cells and to the formation of circulating immune complexes (Fig. 2) [42–44]. The deposition of immune complexes in tissues and the consequent activation of the complement cascade, together with lymphocyte cytotoxicity and the pro-inflammatory effect of type I interferon (IFN-I) and other cytokines (e.g. tumoral necrosis factor alpha, interleukin 6), represent the major determinants in the development of end-organ damage, particularly in the kidney (Fig. 2) [42, 44, 45].

The histological classification currently used for early-onset LN is the same as that used for adult patients, i.e. the ISN/RPS classification [46]. The panel of expert nephropathologists defined criteria for (i) adequacy, (ii) which histological techniques are required to reach a diagnosis of LN, and (iii) which combinations of findings are required to meet the criteria for a specific class of LN, identifying six such classes.

An adequate kidney sample should include at least 10 glomeruli, but whether globally sclerosed glomeruli should be counted is not specified. Required techniques to establish a diagnosis are light and immunofluorescence microscopy, whereas electron microscopy, usually very informative, is not mandatory. Detection of IgG and C3 on immunofluorescence microscopy is a required minimum, whereas full house (i.e. IgG, IgA, IgM, C3, and C1q positivity) is expected in most instances. This means that restricted positivity for IgA and/or IgM should alert the pathologist of other aetiologies than LN. Histological mimics of LN are glomerulonephritis in which a full house pattern can be identified despite the absence of clinical and laboratory findings attributable to SLE. The recognition of such entities led to the definition of ‘non-lupus full house nephropathy’ [47]. Only a few patients (6%) receiving this diagnosis develop SLE during subsequent follow-up, whereas the others can be divided in two distinct pathogenic groups, i.e. idiopathic vs secondary forms of non-lupus full house nephropathy, where secondary forms represent about one half of these cases and are mostly infection-related (e.g. HIV, Bartonella, Schistosoma, HBV, parvovirus B19), but drug-related disease and concurrent glomerulopathies (e.g. membranous nephropathy, IgA-nephropathy) have also been documented [47].

The classification lists a series of patterns entirely based on glomerular lesions, i.e. the mesangial pattern (mesangial expansion due to hypercellularity or matrix accumulation), the endothelial pattern (endocapillary hypercellularity leading or not to a membranoproliferative or mesangiocapillary pattern), and the epithelial pattern (membranous nephropathy). Curiously, there is no reference to a ‘vasculitic’ pattern, i.e. extracapillary glomerulonephritis, although crescents are listed as active lesions and are part of the classification.

The classification also provides a list of specific lesions, mostly glomerular, which, if absent or present in isolation or combined, allow for a specific class diagnosis to be reached. These lesions include active lesions, namely: wire loops, hyaline thrombi, endocapillary proliferation, karyorrhexis, fibrinoid necrosis, rupture of glomerular basement membrane, and extracapillary proliferation; and chronic lesions, i.e. segmental or global glomerulosclerosis, fibrous adhesions, and fibrous crescents.

Class I is defined as minimal mesangial LN, i.e. mesangial deposits on immunofluorescence and no changes on light microscopy. Class II is defined as mesangial proliferative LN, i.e. mesangial deposits on immunofluorescence and a mesangioproliferative pattern on light microscopy, due to mesangial hypercellularity or mesangial matrix expansion, or both. Class III and IV are defined as focal (III) and diffuse (IV) proliferative LN, i.e. any combination of the afore-mentioned active lesions, with a class III diagnosis if such lesions are focal (i.e. they involve <50% of all glomeruli) and a class IV diagnosis if such lesions are diffuse (i.e. they involve >50% of all glomeruli). A special case of class IV is that of global and diffuse subendothelial deposits with little or no proliferation. Class V is membranous LN. Class VI corresponds to the so-called ‘advanced-stage LN’, i.e. with >90% of glomeruli showing global glomerulosclerosis and no evidence of active glomerular disease. This diagnosis implies that a clinical or pathological evidence that such glomerulosclerosis is attributable to LN exists, i.e. the prior evidence on a previous kidney biopsy of ongoing active LN.

A diagnosis of mixed classes is possible exclusively when proliferative lesions and a membranous pattern coexist (i.e. III or IV plus V), with an important caveat: to reach a diagnosis of combined class V and class III or IV, membranous lesions must be global (i.e. involving >50% of the glomerular tuft) and diffuse. On the other hand, to reach a diagnosis of pure class V LN, membranous lesions can be focal or global, segmental, or diffuse. The presence of mesangial hypercellularity or matrix expansion in a case of proliferative or membranous LN is frequent but a diagnosis of mixed class II and III/IV or V is not permitted by the classification. The rationale of such a choice is based on two principles: it is postulated to exist a sequence in immune-complex deposition, the mesangial deposits being the earliest to appear, such that a diagnosis, for instance, of mixed mesangial and membranous LN is not informative; proliferative classes are the most clinically aggressive, therefore if proliferative lesions are present in the setting of membranous LN, this must be stated clearly in the histological report since it carries key management implications.

In 2018, a revision of the ISN/RPS classification proposed the elimination of sclerosing classes (not mentioned here since they ‘did not make it’ into nephropathology clinical practice, being perceived as confusing and controversial), and the systematic adoption of slightly modified NIH activity and chronicity indices for all classes [48]. The proposed modified NIH activity index includes scoring of the following lesions: endocapillary hypercellularity, karyorrhexis, fibrinoid necrosis, cellular/fibrocellular crescents, hyaline deposits, and interstitial inflammation. The proposed modified NIH chronicity index includes the scoring of total glomerulosclerosis, tubular atrophy, interstitial fibrosis, and fibrous crescents. Such indices were originally meant to account for clinicopathological correlations [49], with inconsistent findings across later studies [50].

**Figure 3:**
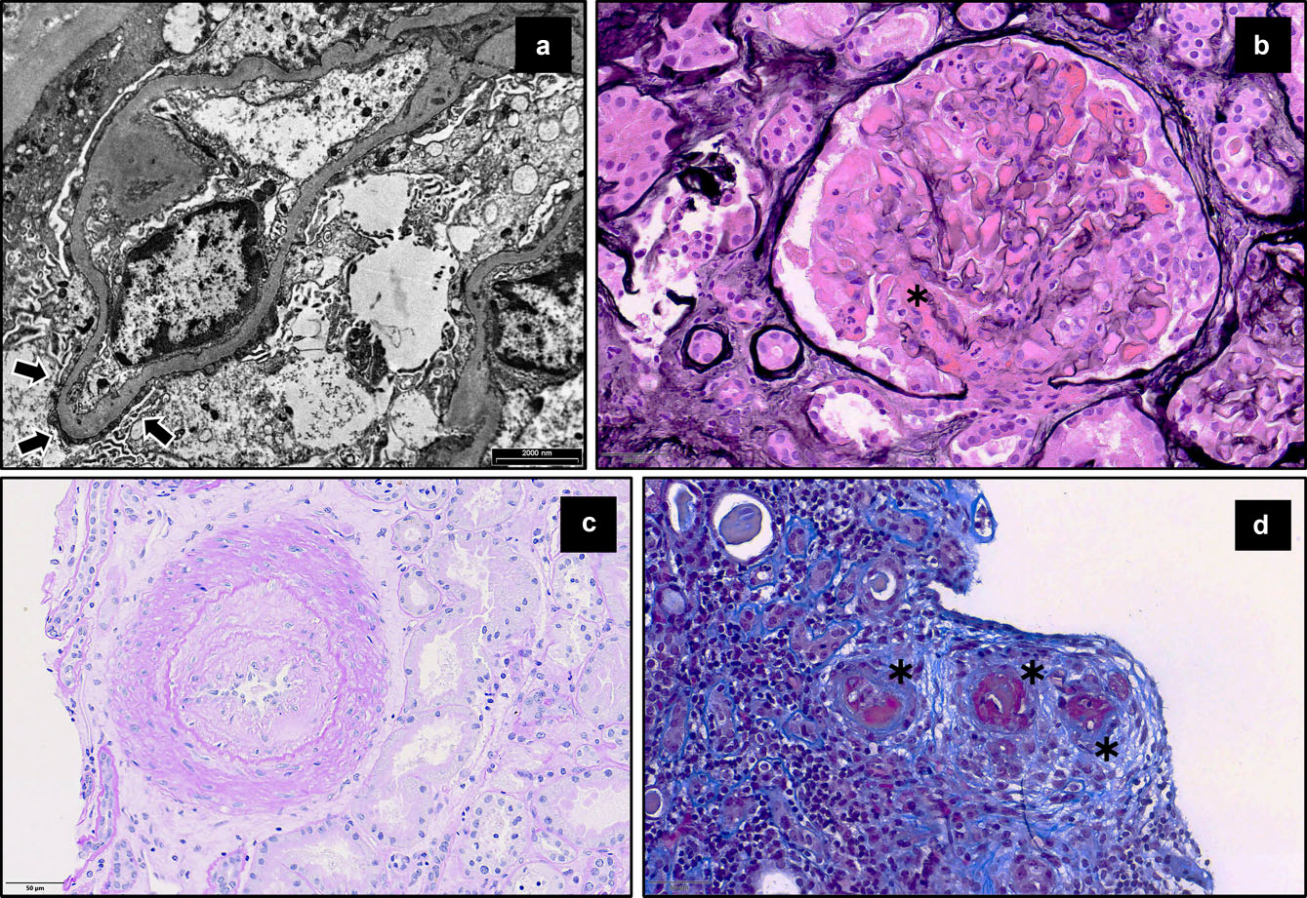
Kidney histological findings of lupus not included in the ISN/RPS classification. (**a**) Lupus podocytopathy. Ultrastructural image of a glomerular capillary loop showing complete podocyte foot process effacement (arrows). This came from a patient with new-onset nephrotic syndrome, systemic lupus manifestations and a normal appearance of kidney histology on light microscopy. (**b**) Thrombotic microangiopathy. Jones’ methenamine-silver-stained slide showing a glomerulus with global intracapillary proliferation and a mixed fibrin and cellular thrombus in the hilum (asterisk). Thrombotic microangiopathy in this patient coexisted with a diagnosis of international society of nephrology and renal pathology society (ISN/RPS) class IV LN. Antiphospholipid antibodies were negative. (**c**) Antiphospholipid nephropathy. Periodic acid Schiff-stained slide showing a large arteriole with near-complete occlusion due to massive fibrointimal hyperplasia with sparse cells admixed in the proliferating subintimal matrix. Such a lesion is typical of antiphospholipid nephropathy and coexisted in a patient with a diagnosis of ISN/RPS class II LN. Antiphospholipid syndrome was secondary to lupus. (**d**) Lupus vasculopathy. Masson's trichrome stained slide showing an arteriole (asterisks) completely occluded with massive subendothelial fuchsinophilic deposits, which were positive for IgG, IgA, IgM, C3, and C1q on immunofluorescence, consistent with a diagnosis of lupus vasculopathy. This patient had a concomitant ISN/RPS class III proliferative LN.

The renal histopathological spectrum of LN, however, includes several non-strictly glomerular entities not included in the ISN/RPS classification, namely: lupus podocytopathy [51], thrombotic microangiopathy (TMA) [52], lesions attributable to secondary antiphospholipid syndrome (i.e. antiphospholipid nephropathy) [53], and lupus vasculopathy [54] (Fig. 3).

While pharmacological trials and treatment recommendations are based on the ISN/RPS classification [55], many of the just-mentioned histopathological entities probably carry prognostic and management implications that require further investigation. It is worth mentioning that at least for TMA there is mounting evidence of an association with unfavourable kidney outcomes [56].

## MONOGENIC LUPUS

Monogenic SLE accounts for 7%–10% of all cases of early-onset SLE and denotes a spectrum of conditions caused by high-penetrance pathogenic variants in a single gene, which are dominantly or recessively inherited. These alterations affect the genes involved in the complement system (e.g. *C1Q, C2, C4A*, and *C4B*), in extracellular (e.g. *TLR7, DNASE1L3*) and intracellular (e.g. *TREX1, RNASEH2B*) nucleic acid sensing and processing pathways, and occasionally RAS signalling (e.g. *KRAS, NRAS*) and in different metabolic pathways (e.g. *RAG1, RAG2*) [57]. Some of these monogenic forms (particularly those linked to complement mutations) have clinical phenotypes that are strikingly similar to those of sporadic SLE. In other monogenic conditions, the clinical phenotypes only partially overlap with that of sporadic SLE; these include the type I interferonopathies (T1Is), which are due to mutations of genes involved in the regulation of the IFN-I pathway, and lead to its constitutive hyperactivation. Genetic abnormalities in T1Is include loss-of-function mutations affecting genes encoding nucleases such as RNASEs and DNASEs, gain-of-function mutations of genes encoding dsRNA sensors (e.g. *MDA5* and *RIG-I*) or other proteins such as STING, an adaptor signalling molecule of the DNA sensing pathway [58]. Interestingly, sporadic SLE as well is characterized by functional impairment in nucleases such as DNASE1L3 and an excessive load of poorly digested nucleic acids [59, 60].

The most common T1Is that feature SLE phenotypes comprise DNASE1L3 and DNASEII deficiencies, COPA (coatomer subunit-α) syndrome, Aicardi–Goutières syndrome (AGS), and STING-associated vasculopathy with onset in infancy (SAVI). These entities may also show renal involvement, with histopathological patterns usually characterized by immune-complex mediated glomerulonephritis, encompassing proliferative (e.g. endo- or extracapillary, membranoproliferative) and non-proliferative (e.g. membranous nephropathy) pathologies, but also TMA and podocytopathies [61]. These glomerular lesions are linked to IFN-I, which may induce direct tissue damage and promote glomerular inflammation. In keeping with this hypothesis, in several T1Is with renal involvement we observed an increased glomerular expression of the IFN-I-induced protein MXA, which co-localized with markers of glomerular endothelial and inflammatory cells in proliferative forms (due to DNASE1L3 deficiency), of extra-glomerular endothelial cells in TMA (secondary to adenosine-deaminase 2 deficiency), and of parietal epithelial cells in collapsing glomerulopathy (secondary to RNASEH2B deficiency). The topographic correspondence between MXA expression and glomerular lesions strengthens the hypothesis that IFN-I responses have a pathogenic role in kidney damage [61–63].

Overall, LN secondary to T1Is or, more broadly, to monogenic conditions, should be suspected in patients who (i) have an early age at disease onset (often <10 years), (ii) are less frequently female than in sporadic SLE, (iii) have familial aggregation (such as in autosomal dominant conditions like COPA), (iv) have atypical manifestations (e.g. basal ganglia calcifications and central neurological syndromes as in AGS, severe interstitial lung disease as in SAVI, severe chilblains as in *TREX1*-related disease), (v) show positivity for both SLE-related autoantibodies (anti-nuclear, anti-ENA, and anti-dsDNA) and other autoantibodies such as anti-neutrophil cytoplasmic antibodies, and (vi) who are refractory to conventional immunosuppressive therapies. Recognizing that LN is secondary to T1Is also has important therapeutic implications, since these conditions may require therapies that specifically target the IFN-I pathway, such as janus kinase (JAK) inhibitors or anti-IFNalpha receptor antibodies, such as anifrolumab [64].

## TREATMENT

The treatment of early-onset LN is in some ways similar to that of adult-onset LN. Its main goals are to achieve renal remission, prevent disease flares, and limit the progression of kidney damage towards chronic kidney disease (CKD). Although clinical presentation is often more aggressive, the treatment approach should always take in consideration some critical issues that are unique to paediatric patients, such as growth, fertility, and long-term sequelae, both disease- and treatment-related (see the following paragraph).

The choice of the induction treatment protocols mainly depends on the histopathological characteristics displayed at the kidney biopsy. The use of corticosteroids (CS) and other immunosuppressive agents, possibly associated with renin–angiotensin–aldosterone system inhibitors, is often guided by the extent of proteinuria (i.e. low-grade vs nephrotic) and the simultaneous presence of extrarenal manifestations. The use of hydroxychloroquine, unless contraindicated, is recommended also in paediatric patients [65, 66].

In patients with class III or IV LN, regardless of the coexistence of a membranous component, guidelines suggest the use of CS in combination with mycophenolate mofetil or cyclophosphamide [55, 65, 66]. As for adults, therapy with rituximab is reserved to patients who show a poor response to standard treatments [65, 66]. Several studies investigated the role of rituximab in paediatric patients with SLE, some of them focusing on LN, and demonstrated its effectiveness not only in the induction phase, but also in maintaining remission [67–69]. A multi-targeted regimen combining CS, mycophenolate and the calcineurin inhibitor tacrolimus also proved efficacious in children with LN who were refractory to first-line induction therapies [70].

The recommended maintenance therapies for classes III and IV are low-dose CS in combination with mycophenolate or azathioprine, which should be carried on for at least 2 years, eventually proceeding to a progressive tapering of all immunosuppressants based on the patient's clinical evolution (i.e. stable remission vs frequent relapses) and the severity of the disease at onset [55, 65, 66].

In patients with a diagnosis of class V LN, the extent of proteinuria and the presence of extrarenal manifestations are the main elements driving the therapeutic decisions: in addition to hydroxychloroquine and renin–angiotensin–aldosterone system inhibitors, CS and other immunosuppressive agents (i.e. mycophenolate, cyclophosphamide, calcineurin inhibitors, rituximab, or azathioprine) are routinely used, particularly in patients with nephrotic-range proteinuria [65, 66].

In case of refractory disease, or at the occurrence of flares, along with the increase of the ongoing CS dose, the switch to another immunosuppressant should be considered. However, before any treatment modification, clinicians should always rule out non-adherence to prescribed therapy, which is a very frequent issue, particularly during adolescence [65].

Conventional therapy has recently been integrated with the B-cell modulating drug belimumab, of which FDA approval for the use in paediatric patients with SLE came in 2019. Belimumab is a monoclonal antibody directed against BAFF, a molecule involved in the activation and differentiation of B cells [71]. Randomized clinical trials investigating its efficacy in combination with conventional induction treatments showed promising results [72–75]. Belimumab was administered in combination with mycophenolate and a significant decrease in proteinuria was observed [76]. In adult patients, guidelines recommend the use of belimumab in combination with mycophenolate or low-dose cyclophosphamide in the induction phase of patients with class III/IV LN, but also in case of disease flares, partial response to conventional induction treatment. Belimumab can also be used for remission maintenance in combination with CS and mycophenolate [66].

Given the rigorous vaccination schedule planned for children and adolescents, it is important to remember that patients that are undergoing treatment for LN can be administered non-live vaccines (e.g. against tetanus, hepatitis A and B, meningococcus, pneumococcus, human papillomavirus, influenzavirus, and SARS-CoV2), whereas the administration of live attenuated ones (e.g. against Varicella–Zoster virus, measles, mumps, rubella, and yellow fever) should be avoided [77].

## OUTCOMES AND LONG-TERM MANAGEMENT

Unfortunately, data on long-term outcomes in patients with early-onset LN are limited, mainly because most studies provide short follow-up periods [78–81]. Moreover, there is a high variability of results, partially depending on the treatment era, but also geographic and socio-economic factors.

To date, treatment success in patients with early-onset LN remains suboptimal, with only 50%–79% of children obtaining a complete renal response after a 2-year treatment course following kidney biopsy [82]. Given this high rate of treatment failure, it is foreseeable that a high number of early-onset LN patients will progress to a certain degree of CKD. Available data show that up to 14% of them develop end-stage kidney disease (ESKD) after a median follow-up of 20 years [83]. The absence of response to first-line therapies, the severity of kidney impairment at presentation, and the occurrence of kidney flares are all demonstrated predictors of adverse renal outcomes [80, 81, 83–85]. Kidney flares are frequent in patients with early-onset LN: indeed, 25%–60% of children experience at least one relapse over their clinical course [80, 82, 85–89]. Each flare has a negative impact on the residual kidney function and requires a strengthening of immunosuppressive therapies, further increasing the cumulative toxicity determined by such treatments. Therefore, it is not surprising that disease flares are not only associated with an increased risk of CKD and ESKD, but also with a higher incidence of treatment-related adverse events, such as infections and osteopenia [80, 82, 85–89]. An increased risk of disease relapse was observed in patients with a younger age at LN diagnosis, those treated with azathioprine as maintenance therapy, and those achieving only partial or no response to first-line treatment [85]. Another crucial aspect in the management of early-onset LN, possibly contributing to treatment failure and increased frequency of renal and extrarenal flares, is treatment non-adherence. This issue is particularly relevant in adolescent patients [90]; therefore, treatment non-adherence should always be ruled out before any further decision on therapy modifications [65].

Given the importance of obtaining a complete response and a decrease in the rate of kidney flares, the assessment of disease activity becomes a key element in the management of these patients. This evaluation relies on different internationally validated scores, such as the SLEDAI, and the British Isles Lupus Assessment Group (BILAG) score [91]. European evidence-based recommendations state that all paediatric patients with SLE should undergo regular disease activity evaluations, using either the SLEDAI 2000 (SLEDAI-2 K) or the paediatric BILAG index 2004 (pBILAG-2004) score [65]. A satisfactory treatment regimen should at least aim at obtaining a sustained lupus low disease activity state (LLDAS) [92], a parameter recently introduced into clinical practice, which is associated with a lower number of disease flares, and a lower risk of disease-related damage [93]. This score is widely validated also in the paediatric population [94–96].

As for disease activity, cumulative organ damage can be evaluated using a validated clinical score, the Systemic Lupus International Collaborating Clinics/American College of Rheumatology Damage Index, which has been implemented with two specific domains for paediatric patients: growth failure and delay in secondary sexual characteristics [97]. European evidence-based recommendations advise the use of this score for annual assessment of cumulative damage in paediatric patients with SLE [65]. The high burden of chronic morbidity is a consequence of severe acute phases of the disease and of a possible persistent degree of disease activity, but drug-related toxicity clearly plays a significant role. After a median follow-up of 20 years, 62% of early-onset LN patients show some degree of chronic damage, mainly involving the kidneys, the musculoskeletal, and the neurological systems [83]. With regards to CS toxicity, this is reported in >40% of patients, who can develop Cushingoid changes (30%), osteopenia (32%–41%), avascular osteonecrosis (7%–10%), and cataract (5%) [84, 98–100]. Another relevant aspect in patients with early-onset LN is represented by growth impairment, which is reported in up to 78% of the cases [98]. A cumulative dose of CS >10 g or >230 mg/kg and an early age at disease onset (i.e. before 12 years of age) are recognized risk factors for growth impairment and pubertal development [77, 101]. Early-onset severe cardiovascular events are also described, with cerebrovascular accidents and myocardial infarction occurring at a median age of 20 and 39 years, respectively [83]. The risk of fertility impairment is increased by the use of cyclophosphamide, particularly in male individuals who received a cumulative doses >7.5 g/m^2^ and in female patients who received a cumulative dose >10–15 g/m^2^ [102].

Infections represent the most common treatment-related complication in patients with early-onset SLE, and severe infectious episodes in the context of an aggressive systemic disease are the first cause of death in these patients, with overall mortality rates being highest in children who have a very early disease onset (i.e. before 6 years of age) [17]. Five-year mortality rates range from 4% to 23% [103], mainly depending on geographic and socio-economic factors. Despite the availability of effective immunosuppressive therapies, no further improvement in patient survival has been observed in the last decades, compared to previous years [103].

## CONCLUSIONS

Early-onset LN is an often-severe condition, commonly associated with other aggressive manifestations of SLE. Histopathological evaluation of kidney biopsies is a key determinant of treatment choices, along with a careful assessment of the clinical phenotype of the disease. Monogenic forms of SLE should be considered because of their peculiar clinical presentation and the potential treatment implications. In the management of early-onset LN, immunosuppressive therapies should be used in combination with preventive strategies aimed at limiting the possible disease-related and treatment-related long-term complications, some of which are particularly important as they impact on the growth and overall health of these young patients.

## Data Availability

No new data were generated or analysed in support of this research.
